# Measurement of maximum tongue protrusion force (MTPF) in healthy young adults

**DOI:** 10.14814/phy2.14175

**Published:** 2019-07-10

**Authors:** Jayoung Kim, Karen Hegland, William Vann, Richard Berry, Paul W. Davenport

**Affiliations:** ^1^ Department of Speech, Language and Hearing Sciences University of Florida Gainesville Florida; ^2^ Department of Physiological Sciences University of Florida Gainesville Florida; ^3^ Department of Medicine University of Florida Gainesville Florida

**Keywords:** Snoring, speech/swallow disorders, tongue protrusion force, upper airway patency

## Abstract

We propose that tongue protrusive strength and tone may be related to upper airway patency, and when protrusive strength is reduced, individuals are at higher risk of developing sleep apnea, or speech/swallow disorders. The goal of the current study was to determine normative values of maximum tongue protrusion force (MTPF) in healthy young adults, using a unique newly developed device. We hypothesized that MTPF would be greater in males than in females. One hundred and one healthy young adults (mean age: 22.99 years; male: 23, female: 78) participated in this study. The subjects pushed their tongue forward against the device’s piston (protrusion) as hard as possible for 2–5 sec and MTPF was recorded in Newtons (N). A minimum of 5 MTPF measurements were obtained with 1–2 min rest between measurements. The average MTPF for all subjects was 15.4 N (SD: ±3.8), with a range of 8–29. The male average MTPF was higher than female (17.8 N, SD: ±3.7 vs. 14.7 N, SD: ±3.5; *P *= 0.001). There was no significant difference for age between males and females; males had significantly greater height and weight. The results demonstrate our novel device can effectively measure tongue protrusive force in healthy young adults. This study provides normative values for MTPF, and identified significant tongue protrusion strength differences between males and females.

## Introduction

The genioglossus (GG) muscle is a fan‐shaped extrinsic tongue muscle spanning the anterior wall of the oropharynx (Fregosi and Fuller, 1997). The GG is one of the primary lingual muscles responsible for tongue protrusion. Tongue protrusion is important for speech, swallow, and breathing. The GG and associated muscle activity maintains airway patency under negative‐pressure conditions (Wheatley et al. 1993). GG electromyogram studies show that GG is active during the inspiratory phase of respiration, and healthy individuals exhibit rhythmic activation of the GG with inspiration when in the upright posture (Mortimore and Douglas, 1996; Ono et al. 1996; O’Connor et al. 2007; McSharry et al. 2012). However, GG becomes more collapsible with rising upper airway resistance during sleep, and the activation of GG increases as inspiration efforts increase to enlarge the volume of the airway (Ono et al. 1996). Therefore, contraction of GG plays an important role in maintaining upper airway patency, preventing posterior tongue displacement and upper airway closure.

During swallowing, the tongue manipulates the bolus for mastication and transport of the bolus to oropharynx. Contraction of the GG fixes the anterior dorsal surface of the tongue against the hard palate (Matsuo and Palmer, 2010). The GG in coordination with associated muscles influence tongue location and posture for midline grooving and protruding the tongue body, which is integral in the production of several vowels and consonants (Hiiemae and Palmer, 2003). Reduced tongue protrusion force and tone can result in sleep apnea (Young et al. 2002; Cheng et al. 2008), disordered speech (Trawitzki et al. 2011) and disordered swallowing (Hewitt et al. 2008; Utanohara et al. 2008; Hori et al. 2009).

To date, most studies of tongue strength have investigated the vertical force produced (Robin and Luschei, 1992; Utanohara et al. 2008; Trawitzki et al. 2011; Adams et al. 2013; Adams et al. 2014). The primary muscles involved in vertical movement of the tongue are the styloglossus for raising and retracting the tongue, and palatoglossus for pulling the tongue back to grove (Hiiemae and Palmer, 2003; Cheng et al. 2008). Also, superior/inferior longitudinal muscles are involved in curling the tongue up and down, respectively (Hiiemae and Palmer, 2003; Cheng et al. 2008). Vertical movement of the tongue is important for both mastication and swallowing, and reduced vertical force (an indirect measure of tongue strength) has been associated with disordered swallowing (Lazarus et al. 2000; Stierwalt and Clark, 2002; Clark et al. 2003; Yoshida et al. 2006; Youmans and Stierwalt, 2006). Specifically, researchers have found that reduced vertical tongue strength can result in oral phase swallowing impairment, especially manipulation of a bolus, and reduced contact duration of the tongue base to the pharyngeal wall in oropharyngeal phase.

Despite the known contributions of the tongue protrusion muscles, including the GG, to breathing, speech and swallowing functions, there are very few studies of tongue protrusion force. Mortimore et al. (1999) measured tongue protrusion force in 81 males and 86 females. The results demonstrated that tongue protrusion force was greater in males than in females, and that it decreased with age in both sexes (Mortimore et al. 1999). Araújo et al. (2018) evaluated axial tongue strength to 49 healthy young adults. The results demonstrated a significant increase of tongue protrusion force by training.

Based on the assumption that the measurement of tongue protrusion force is a proxy for GG function, the goal of the current study was to determine normative values of maximum tongue protrusion force (MTPF) in healthy young adults, using a newly developed device. The unique device was designed to be small and portable such that measuring tongue protrusion force could be performed outside of the laboratory, as might be required if it were to be used in a clinic or hospital setting. It was hypothesized that the device would be easy to use and produce consistent measures of force within participants. As well, similar to the previous study by Mortimore et al. (1999), it was hypothesized that MTPF would be greater in males than in females.

## Materials and methods

### Participants

The Institutional Review Board (IRB) at the University of Florida approved all study procedures, and all participants were volunteers who provided verbal and written informed consent (IRB no. 201700324). Participants were healthy individuals, males, and females, aged 18–37 years. Participants with a self‐history of cancer or surgery in the mouth or tongue, sleep apnea, or any neurological disease were excluded from this study. Height and weight were collected for each participant.

### Study device and protocol

**Figure 1:**
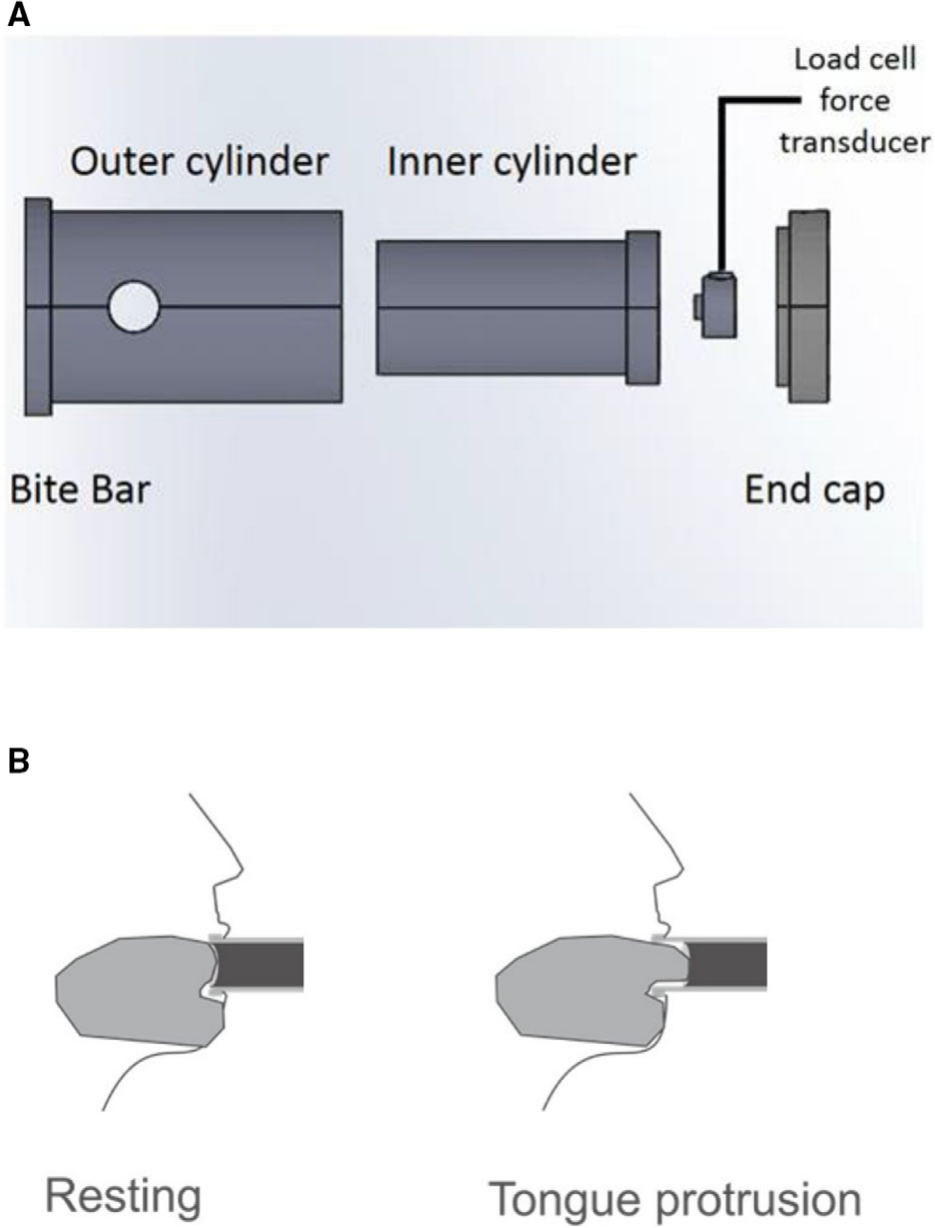
Schematic representation of measuring tongue protrusion force device: (A). The device was a piston in a cylinder with a force transducer at the base of the piston. A strain gauge was inserted between the end of the cylinder and the end of the piston; (B). The bite bar of the device was held by the front teeth, and the tongue extended 0.5–1 cm beyond the teeth to touch the piston. Participants were instructed to push their tongue forward against the piston as hard as possible for 2–5 sec.

**Figure 2:**
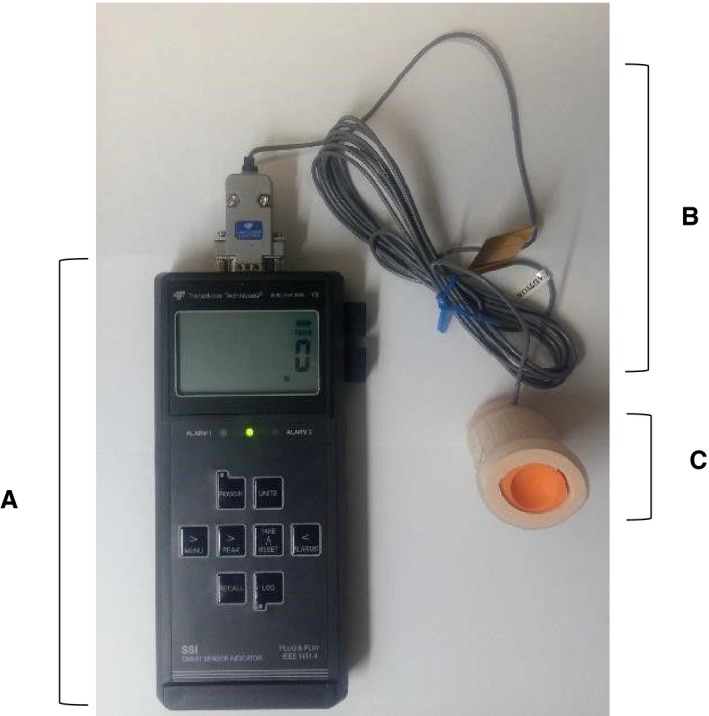
A measuring device: (A) a recording device (B) a connector (C) a piston in a cylinder with a force transducer at the base of the piston.

The tongue protrusion force measurement device was a piston in a cylinder with a force transducer at the base of the piston. A strain gauge was inserted between the end of the cylinder and the end of the piston for isometric force measurement (Fig. 1‐A). The diameter of the inner cylinder was 29 mm (horizontal) and 22 mm (vertical), and that of the outer cylinder was 37.5 mm (horizontal) and 30 mm (vertical). Only one size of this device was used. The device included a connector and a recording device (Prototype device Plug & Play smart portable hand held load cell indicator, model SSI, Transducer Techniques; Fig. 2). The piston does not move because this is an isometric device. The mouthpiece of the device was covered with a waterproof film for each individual. The piston end of the device was inserted into the mouth and held in place by the teeth closing onto the bite bar (Fig. 1‐A). We removed the film at the end of the cylinder for individual use. The subject extended the tip of their tongue (Fig. 1‐B) into the indentation at the end of the piston (Fig. 2).

To measure maximum isometric tongue protrusion force (MTPF), participants were seated in an upright position. The bite bar of the device (Fig. 1‐A) was held by the front teeth, and the tongue extended 0.5–1 cm beyond the teeth to touch the piston. Participants were instructed to push their tongue forward against the piston as hard as possible for 2–5 sec (Fig. 1‐B). Then the participants relaxed for 1–2 min between each trial. Subjects performed 5–10 MTPF efforts, because there was no fatigue effect in MTPF within four trials between males and females including healthy and patient population in the previous study (Mortimore et al. 1999). To identify normative values in healthy young adults, we performed at least five trials. Also, there was significant increase in MTPF from trial 1 to trial 4 (*P* < 0.05), but MTPF from trial 4 to trial 10 was not significantly different (*P* > 0.05), hence we adjusted the protocol to have subjects (*n* = 58) perform 10 trials to obtain a stable MTPF. The MTPF for each effort was recorded in Newtons (N). The MTPF for all efforts were then averaged for each subject.

### Data analysis

Data were analyzed using SPSS statistics 24 (SPSS Corp, Chicago, IL). We used descriptive statistics, including mean and standard deviations, to determine the range of normative values of MTPF and variability of the measure across trials in addition to MTPF maximum values. Analysis of covariance (ANCOVA) was used to determine whether MTPF varied according to sex, with height and weight included as covariates. Pearson correlation coefficients were used to identify the relationship between MTPF and demographic variables including age, weight, and height. Scatter plot of MTPF in each trial was used to analyze variability of MTPF within subjects.

## Results

### Participants

All healthy participants were aged 18–37 years with no self‐reported history of cancer or surgery in the mouth or tongue, sleep apnea, or neurological disease. There were a total of 101 participants (23 males, 78 females; mean age: 22.99 years (SD: ±3.93)) with a range of 18–37 years. Participant demographic information is presented in Table 1.

**Table 1:** Demographic information.

	Sex	N	Mean	SD
Age (years)	Male	23	23.83	3.393
	Female	78	22.74	4.066
Height (cm)	Male	23	178.74	6.489
	Female	78	166.88	6.162
Weight (kg)	Male	23	75.27	9.884
	Female	78	63.90	14.373
BMI (kg/m^2^)	Male	23	23.5	2.8
	Female	78	22.9	4.6
MTPF average (*N*)	Male	23	17.76	3.735
	Female	78	14.72	3.490

BMI, body mass index.

### MTPF

**Figure 3:**
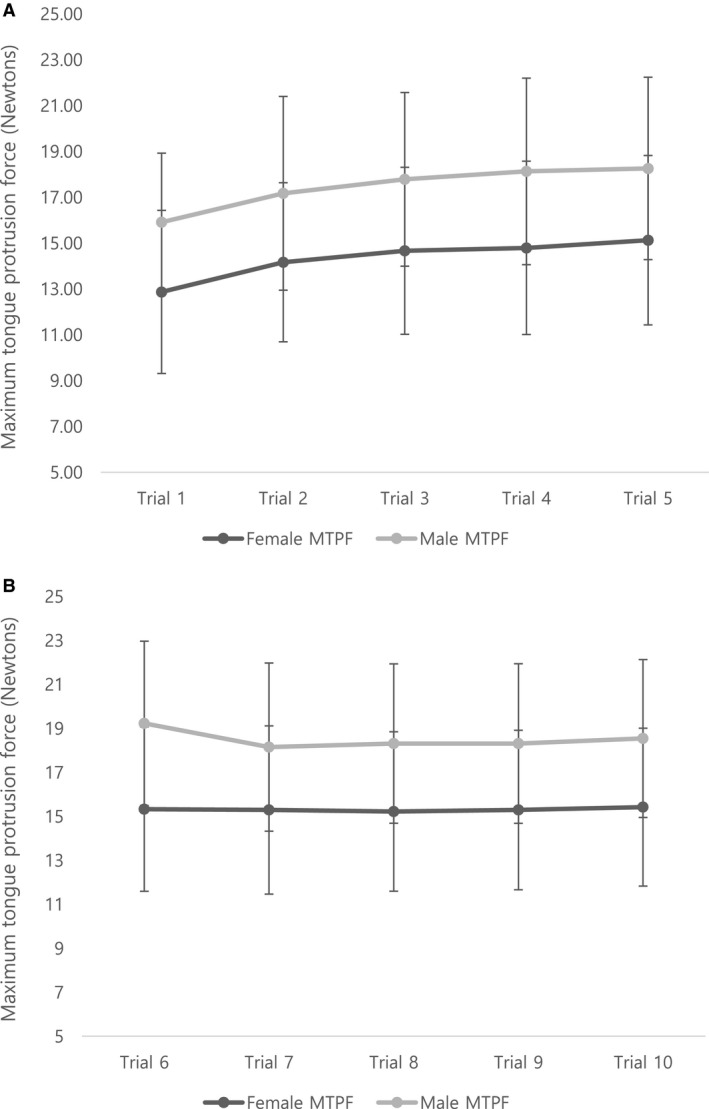
Variability of MTPF within subject: The figure represents the mean MTPF for each trial separated for males and females with the addition of the standard deviation (SD) bars. Figure 3‐A is for all 101 subjects who completed 5 trials, and Figure 3‐B presents 58 of 101 subjects who completed 10 trials. The results show significant increase between trial 1–4 (*P* < 0.05), but MTPF between trial 4–10 is not significantly different (*P *> 0.05). Hence, the average MTPF would be the best to be calculated with trial 4–10.

All subjects were able to perform the MTPF task without reported difficulty or discomfort. A total number of participants who performed trials 1–5 was 101; trials 1–6 was 89; trials 1–7 was 83; trials 1–8 was 70; trials 1–9 was 61; trials 1–10 was 58. The average MTPF for all subjects was 15.4 N (SD: ±3.8), with a range of 8–29 N. The average MTPF was significantly higher in males (mean MTPF* *= 17.8, SD ± 3.7 N) than in females (mean MTPF* *= 14.7, SD ± 3.5 N) after covarying for height and weight (*F *= 9.781; df* *= 1; *P *= 0.002; Table 1). The maximum MTPF produced by the subjects was significantly higher for males (mean MTPFmax* *= 19.43, SD ± 4.26 N) than for females (mean MTPFmax* *= 16.30, SD ± 3.50 N). Figure 3 presents the variability of the task across the 10 trials. Figure 3 presents the mean (±SD) MTPF for each trial separated for males and females. Figure 3‐A provides the mean (±SD) MTPF for all 101 subjects who completed 5 trials. Figure 3‐B presents the mean (±SD) MTPF for the 58 of 101 subjects who completed 10 trials. The results show significant MTPF increase between trials 1 and 4 (*p* <.05), but MTPF between trials 4 and 10 is not significantly different (*P*> 0.05).

**Figure 4:**
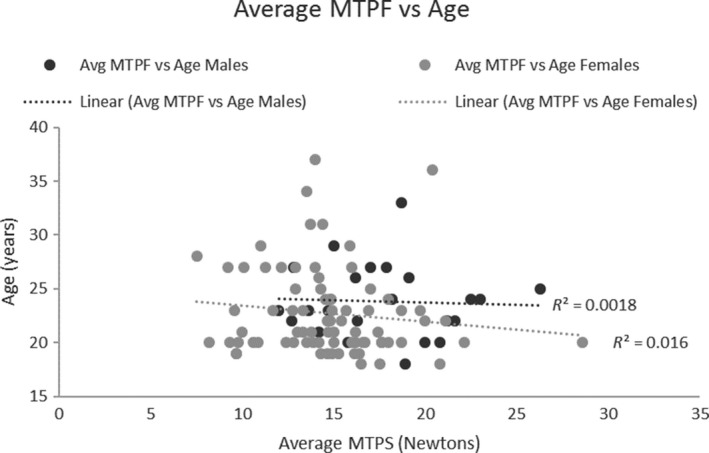
MTPF vs Age scatter plot: MTPF had no significant relationship with age.

**Figure 5:**
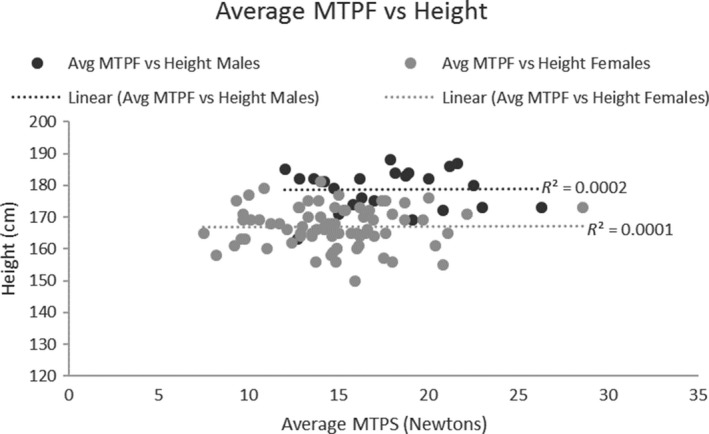
MTPF vs Height scatter plot: MTPF had no significant relationship with height.

**Figure 6:**
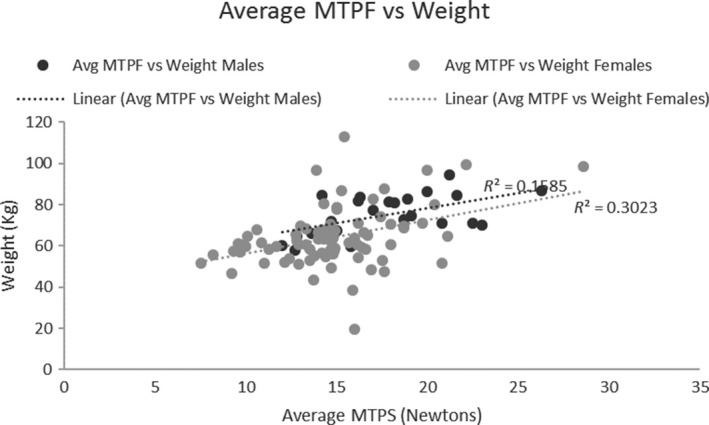
MTPF vs Weight scatter plot: There was a relationship for males, and females between MTPF and weight.

A correlation analysis showed that the mean MTPF was not significantly correlated with age (Male: *r *= 0.04, Female: *r *= 0.13; Fig. 4), or height (Male: *r *= 0.01, Female: *r *= 0.01; Fig. 5) for males and females. There was a weak but significant correlation between MTPF and weight for both males and females (Male: *r *= 0.40, *P *= 0.007; Female: *r *= 0.55, *P *= 0.000; Fig. 6).

## Discussion

The aim of the study was to determine normative values of MTPF, an indirect measure of tongue protrusion strength, in a cohort of self‐reported healthy young adults, using a newly developed isometric tongue protrusion force device. The device was tolerated well and the procedure was completed in all participants. Study results provide normative values for MTPF, and identified significant MTPF differences between males and females.

The results of the current study are in agreement with the Mortimore et al. (1999) study in that the MTPF values for males was significantly higher than females; however, the overall MTPF for both males and females was lower in the current study versus the Mortimore et al. (1999) study. Specifically, the average MTPF for males in the Mortimore study was 26.3 N compared to only 17.8 N in the current study. Similarly, MTPF for females in the former study was 19.9 N, versus 14.7 N in the current study. This is likely due to device and methodological differences. For this study, MTPF was determined by averaging the values produced over 10 tongue protrusion trials. In the Mortimore study, MTPF was reported as the highest value of only two trials. In this study, we presented 5–10 trials to the subjects and found an increasing MTPF for the first 3 trials, plateauing for trials 4–10. The MTPF maximum produced for each subject within the 10 trials increases to 19.43 N, SD: ±4.26 in males and 16.30 N, SD: ±3.50 in females compared to averaged MTPF (males: 17.8 ± 3.7 N; females: 14.7 ± 3.5 N). However, the MTPF maximum in the present study is slightly lower than Mortimore et al (1999), these differences are likely attributable to the use of a different device, positioning of the tongue, number of protrusion efforts, duration of the protrusion effort, and subject age range.

The recent study of Araújo et al. (2018) evaluated axial tongue strength with Forling, which is a portable instrument, in healthy young adults (19 males, 30 females; aged 18–25 years). The authors measured maximum and mean tongue force in three days at intervals of 7 ± 2 days. Three measurements were performed on each day. The mean maximal value on the first day was 16.82 N (SD: ±5.72), the second day was 17.53 N (SD: ±5.22), and the third day was 19.26 N (SD: ±5.22). This study provided values without sex distinction, but overall tongue protrusion force 16.82 N on the first day in this study was slightly higher than 15.4 N in the current study. This also may be due to a methodological difference and the use of a different device. Araújo et al. (2018) demonstrated significant increase of tongue protrusion force between the second and third days, suggesting that tongue protrusion force can be trained.

The current study revealed a significant sex difference even when weight and height were included as covariates. Several authors have identified that sex‐based differences of the tongue strength are attributable to differences in height and weight, as opposed to inherent differences based on sex (Mortimore et al. 1999; Stierwalt and Youmans, 2007; Utanohara et al. 2008; Kays et al. 2010; Vanderwegen et al. 2013). Furthermore, tongue strength is related to overall muscle mass (Almeida et al. 2012).

As can be appreciated from Figure 3, both males and females increased force during the first 3 trials, and were fairly consistent for the remaining trials. This can be a learning effect, hence we recommend excluding the first 2–3 efforts from the mean MTPF trial outcome. In addition, this indicates that participants did not fatigue across 10 trials of this maximal isometric task. Even though there are limitations of the study in the age range (18–37years) and sex ratio (mostly females), we were able to determine the tongue protrusive muscle strength as measured by the MTPF in this cohort of healthy young adults.

Another limitation was the number of trials each individual completed. As the study progressed, we determined that 10 trials is preferred and encouraged our participants to complete 10 MTPF efforts. Our results revealed that both genders increased force during the first 3 trials with fairly consistent force for the remaining trials, and no fatigue effect across 10 trials. Another limitation is using this device requires intact dentition including dentures to secure the device through a bite bar. Therefore, edentulous individuals, or those with sparse dentition could be inappropriate to use this device. Furthermore, the amount of bite closure force required to secure the bite bar and range of motion of tongue might influence the MTPF values. The anatomical limitations in this study need to be investigated in the future.

For future studies, it will be important to determine whether older adults, various patient populations and/or fatigue during the task may indicate reduced endurance of the tongue protrusion muscles. Therefore, to identify MTPF values for healthy older adults may be the next step to compare “normal” MTPF values to those of various patient populations. It will also be important to determine if, like vertical tongue force, reduced protrusion force is associated with functional deficits such as swallowing and speech disorders, or even sleep apnea. Even though individuals have functional deficits, measurement of MTPF may not be sufficient to reveal the impairment because of functional tongue protrusion strength reserve. Understanding MTPF values with overall disease state and other factors such as age and sex would be essential. Finally, we consider to use MTPF values relevant as a screening tool to identify normal versus disordered functions in clinical conditions. If MTPF is reduced, strength training of tongue protrusion could be a novel target for treatment of patients.

## Conflict of Interest

None declared.
